# Metformin for neurocognitive dysfunction in schizophrenia: a systematic review

**DOI:** 10.3389/fpsyt.2024.1540153

**Published:** 2025-01-20

**Authors:** Zhen-Juan Qin, Zhan-Ming Shi, Li-Juan Li, Xin Wei, Hui-Lin Hu, Wei Wei, Zhi-Yuan Xie, Hang-Xi Ji, Yu-Hua Wei, Wei Zheng

**Affiliations:** ^1^ Department of Psychiatry, The Brain Hospital of Guangxi Zhuang Autonomous Region, LiuZhou, China; ^2^ Department of Psychiatry, Chongqing Jiangbei Mental Health Center, Chongqing, China; ^3^ Department of Neurology, The First Affiliated Hospital of Hainan Medical University, Haikou, China; ^4^ Department of Psychiatry, The Affiliated Brain Hospital, Guangzhou Medical University, Guangzhou, China; ^5^ Department of Psychiatry, Key Laboratory of Neurogenetics and Channelopathies of Guangdong Province and the Ministry of Education of China, Guangzhou Medical University, Guangzhou, China

**Keywords:** metformin, schizophrenia, neurocognitive dysfunction, systematic review, efficacy

## Abstract

**Background:**

The efficacy and safety of metformin for addressing neurocognitive dysfunction in schizophrenia remain inconclusive. This systematic review evaluates the evidence from randomized controlled trials (RCTs) on the effects of metformin on neurocognitive function in patients with schizophrenia.

**Methods:**

A comprehensive search of Chinese databases (WanFang, Chinese Journal Net) and English databases (PubMed, EMBASE, PsycINFO, and Cochrane Library) was conducted to identify RCTs assessing metformin’s impact on neurocognitive outcomes in schizophrenia.

**Results:**

Four RCTs involving 271 patients with schizophrenia were included. Three RCTs (75%) demonstrated significant improvements in neurocognitive function with metformin compared to controls, as assessed by the MATRICS Consensus Cognitive Battery, Repeatable Battery for the Assessment of Neuropsychological Status, and Mini-Mental State Examination, but not the Brief Assessment of Cognition in Schizophrenia. Two RCTs (50%) evaluated metformin's effects on total psychopathology and found no significant differences between groups. Adverse events were reported in two RCTs, with inconsistent findings on decreased appetite and diarrhea. Other adverse events and discontinuation rates were comparable between groups.

**Conclusion:**

Preliminary evidence suggests that metformin may improve neurocognitive function in schizophrenia. However, further large-scale, double-blind, high quality RCTs are warranted to validate these findings.

## Introduction

Schizophrenia is a chronic and severe psychiatric disorder characterized by positive and negative symptoms, neurocognitive dysfunction, and social dysfunction (1). Despite its relatively low lifetime prevalence of approximately 1% (2, 3), schizophrenia imposes substantial socioeconomic burdens and contributes significantly to disability worldwide (4). Long-term or lifelong administration of antipsychotics (APs), such as olanzapine and aripiprazole, is essential for preventing disease recurrence (5). However, these medications fail to address neurocognitive dysfunction (6) and may even exacerbate it (7, 8). Thus, there is an urgent need for effective therapeutic strategies targeting neurocognitive symptoms.

Guidelines from the European Psychiatric Association (9) recommend managing neurocognitive dysfunction in schizophrenia through pharmacological interventions, psychosocial strategies, and somatic treatments, which include non-invasive brain stimulation techniques such as transcranial direct current stimulation (tDCS) (10) and repetitive transcranial magnetic stimulation (rTMS) (11). However, these interventions provide only modest improvements in neurocognitive performance (9). Neurocognitive symptoms in schizophrenia often persist despite the resolution of positive symptoms, posing a significant therapeutic challenge (12). Therefore, it is urgent to improve neurocognitive function with effective pharmacological agents.

Metformin, a biguanide hypoglycaemic agent commonly prescribed for type 2 diabetes management (6, 13), has garnered attention for its potential neuroprotective effects. It readily crosses the blood-brain barrier and enhances neurocognitive function through anti-inflammatory mechanisms and improved cerebral energy metabolism (14). Concurrently, neuroimaging modalities such as magnetic resonance imaging and positron emission tomography have implicated neuroinflammation and cerebral metabolic stress in neurocognitive impairment (15, 16). Supporting these mechanisms, animal studies have demonstrated that metformin ameliorates clozapine-induced learning deficits (17) and dizocilpine-induced working memory impairments (18). Additionally, a longitudinal cohort study linked metformin use to improved performance in verbal learning, working memory, and executive function (19). However, randomized controlled trials (RCTs) (20–23) investigating metformin for neurocognitive dysfunction in individuals with schizophrenia and comorbid physical disease (e.g., type 2 diabetes) have reported inconsistent findings.

While previous systematic reviews and meta-analyses (6, 24–26) have focused on the efficacy of metformin in addressing antipsychotic-induced dyslipidemia and weight gain, no systematic review of RCTs has evaluated its effects on neurocognitive function in schizophrenia. To understand the current literature on the role of metformin for neurocognitive effects in schizophrenia, and to provide a more comprehensive and robust basis for clinical application, this systematic review systematically examines the neurocognitive effects of metformin in patients with schizophrenia.

## Method

### Search strategy

A systematic search was conducted in Chinese databases (WanFang and Chinese Journal Net) and English databases (PubMed, Cochrane Library, PsycINFO, and EMBASE) from their inception to August 29, 2024, by three independent authors (ZJQ, ZMS, and LJL). The search terms used in PubMed included: (‘schizophrenia’[MeSH] OR schizophrenic disorder OR schizophrenia OR dementia praecox) AND (‘cognition’[MeSH] OR cognit* OR neurocognit*) AND (‘metformin’[MeSH] OR metformin OR dimethylbiguanidium OR glucophage OR glucovance). Additional articles were identified by manually screening the reference lists of included studies (20–23), relevant reviews (27, 28) and prior meta-analyses (6, 29).

### Selection criteria

Studies were selected based on the Preferred Reporting Items for Systematic Reviews and Meta-Analyses (PRISMA) guidelines (30), following the ***PICOS*** framework: Participants (***P***): adult patients suffering from schizophrenia with or without physical comorbidities, such as type 2 diabetes. Intervention (***I***) versus Comparison (***C***): metformin plus treatment as usual (TAU) versus placebo plus TAU or TAU. Outcomes (***O***): the primary outcome was considered as changes in neurocognitive function assessed using standardized scales (e.g., the Brief Assessment of Cognition in Schizophrenia (BACS) (31), the MATRICS Consensus Cognitive Battery (MCCB) (32), the Mini-Mental State Examination (MMSE) (33) and Repeatable Battery for the Assessment of Neuropsychological Status (RBANS) (34)). Key secondary outcomes were as follows: 1) changes in total psychopathology as measured by standardized scales (e.g., Brief Psychiatric Rating Scale (BPRS) (35) and Positive and Negative Syndrome Scale (PANSS) (36)), 2) adverse events and 3) discontinuation rate. Study design (***S***): published RCTs examining the neurocognitive effects of metformin in adults with schizophrenia, with or without physical comorbidities were included. RCTs focusing on metformin versus acarbose (37), AP-induced hyperprolactinemia (38), weight gain (39), or metabolic syndrome (40) that did not examine neurocognitive effects of metformin were excluded. Furthermore, Case reports/series, animal trials, non-randomized studies, reviews and meta-analyses were also excluded.

### Data extraction

Three investigators (ZJQ, ZMS, and LJL) independently extracted data from each eligible RCT and verified the information. Discrepancies were resolved through discussions with a senior author (WZ). Missing data were requested from the corresponding or first authors via email or phone.

### Study quality assessment

The same three investigators (ZJQ, ZMS, and LJL) independently evaluated the quality of the included studies using both the Cochrane risk of bias tool (41) and the Jadad scale (0–5 points) (42). Studies with a Jadad score of 3 or higher were categorized as ‘high quality’ (43).

## Results

### Study selection

**Figure 1:**
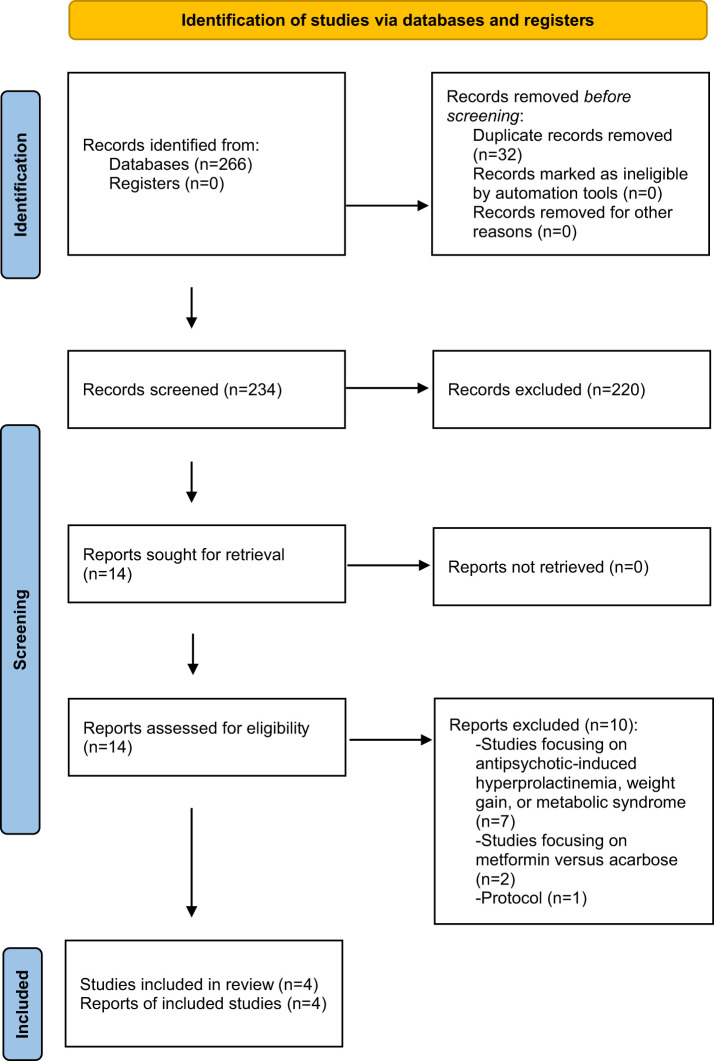
PRISMA flow diagram.

Four RCTs conducted between 2019 and 2023 (20–23) were included in this systematic review. The study selection process is illustrated in **Figure 1**.

### Study characteristics

A total of 271 patients were analyzed across the four RCTs, with 152 participants in the metformin group (750–1500 mg/day) and 119 in the control group. The weighted average age of participants was 34.9 years, and 47.2% of them were male. The trial durations ranged from 8 to 24 weeks. Of the included RCTs, all enrolled patients with schizophrenia and comorbid physical illnesses or high risk of Metabolic syndrome. The detailed characteristics of the studies were summarized in **Table 1**.

**Table 1:** Summary of the characteristics of the included studies.

Study(country)	Number of patients	Trial duration(weeks)	Setting (%)	-Diagnostic criteria Diagnosis (%)	Comorbid physical illnesses or syndrome	Illness duration (years)	Mean age (range) (years)	Sex: Male (%)	Interventions: Mean dose (mg/day) Range (mg/day)Number of patients	Jadad score
Agarwal et al., 2021 (Canada) (23)	30	16	NR	-DSM-5 -SCZ (50.0), SAD (10,0), BD (6.7), psychosis (3.3), paranoid schizophrenia (3.3), multiple diagnosis (26.7)	Type 2 diabetes or prediabetes	8.6	31.6 (17-45)	14 (46.7)	1. Met (M=1500^a^; R=500-1500) + TAU; n=21 2. Placebo + TAU; n=9	5
Shao et al., 2023 (China) (20)	69^b^	24	NR	-DSM-5 -SCZ (100)	High risk of metabolic syndrome	1.8	22.8 (18-65)	13 (18.8)	1. Met (M=1500^c^; R=500-1500) + TAU; n=45 2. TAU; n=24	3
Wang et al., 2019 (China) (21)	100	12	Inpatients (100)	-ICD-10 -SCZ (100)	Type 2 diabetes	5.2	42.4 (NR)	61 (61.0)	1. Met (fixed dose at 1500) + TAU; n=50 2. Placebo + TAU; n=50	2
Xiong et al., 2021 (China) (22)	72	8	NR	-ICD-11 -SCZ (100)	Glucose dysregulation	1.3	37.3 (NR)	40 (55.6)	1. Met (fixed dose at 750) + TAU; n=36 2. TAU; n=36	2

^a^The active drugs of the trial started with a low dosage and added to the target dosage within 14 days.

^b^Number of patients was based on a complete baseline neurocognitive function test.

^c^The active drugs of the trial started with a low dosage and added to the target dosage within 5 days.

Abbreviations: BD, bipolar disorder; DSM-5, Diagnostic and Statistical Manual of Mental Disorders 5^th^ edition; ICD-10, International Classification of Diseases, 10^th^ edition; ICD-11, International Classification of Diseases, 11^th^ edition; M, mean; Met, metformin; NR, not reported; R, range; SAD, schizoaffective disorder; SCZ, schizophrenia; TAU, treatment as usual.

### Assessment of study quality

**Figure 2:**
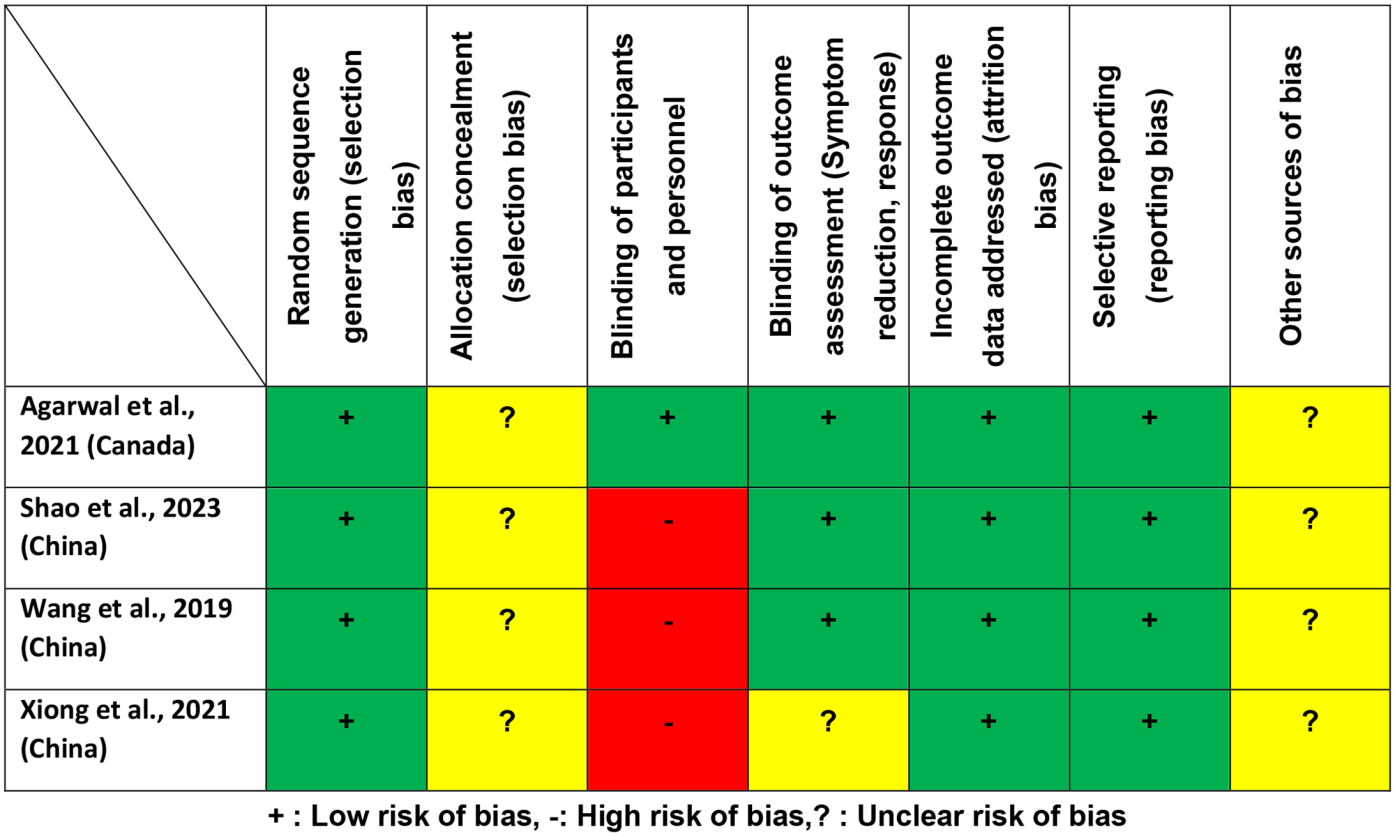
Cochrane risk of bias.

The Jadad scores of the included RCTs ranged from 2 to 5, with two studies (50%) (20, 23) classified as high quality (Jadad score ≥ 3). According to the Cochrane risk of bias assessment (**Figure 2**), all included studies demonstrated a ‘low risk’ for random sequence generation, addressing incomplete outcome data and selective reporting.

### Neurocognitive function

All four RCTs evaluated the neurocognitive effects of metformin in schizophrenia, but their data could not be pooled due to variations in measurement tools. As summarized in **Table 2**, three RCTs (75%, 3/4) demonstrated significant superiority of metformin over controls in improving neurocognitive function, with outcomes assessed using the MCCB (20), RBANS (21), and MMSE (22). However, one RCT (25%, 1/4) reported no significant differences between groups when using the BACS (23).

**Table 2:** Metformin for neurocognitive dysfunction in schizophrenia: neurocognitive function.

Study	Daily dosage (mg)	Assessment scales	Findings
Agarwal et al., 2021 (Canada) (23)	1500	BACS	No significant group differences were found regarding neurocognitive function as measured by BACS.
Shao et al., 2023 (China) (20)	1500	MCCB	Metformin significantly improved composite score, speed of processing, working memory, verbal learning, and visual learning as measured by MCCB when compared to the control group.
Wang et al., 2019 (China) (21)	1500	RBANS	Metformin significantly improved immediate memory, visuospatial skills, language, attention, and neurocognitive total score as measured by RBANS when compared to the control group.
Xiong et al., 2021 (China) (22)	750	MMSE	Metformin significantly improved orientation in time and place, memory registration and recall, attention and calculation, and language as measured by MMSE when compared to the control group.

BACS, Brief Assessment of Cognition in Schizophrenia; MCCB, MATRICS Consensus Cognitive Battery; MMSE, Mini-Mental State Examination; RBANS, Repeatable Battery for the Assessment of Neuropsychological Status.

### Total psychopathology

Two RCTs (50%, 2/4) (20, 23) examined the effects of adjunctive metformin on total psychopathology in schizophrenia. Both studies reported no significant differences between the metformin and control groups (**Table 3**).

**Table 3:** Metformin for neurocognitive dysfunction in schizophrenia: total psychopathology.

Study	Assessment scales	Findings
Agarwal et al., 2021 (Canada) (23)	BPRS	No significant group differences were observed regarding total psychopathology as measured by BPRS.
Shao et al., 2023 (China) (20)	PANSS	No significant group differences were observed regarding total psychopathology as measured by PANSS.
Wang et al., 2019 (China) (21)	NR	NR
Xiong et al., 2021 (China) (22)	NR	NR

BPRS, Brief Psychiatric Rating Scale; NR, not reported; PANSS, Positive and Negative Syndrome Scale.

### Adverse events and discontinuation rate

Two RCTs (50%, 2/4) (20, 23) reported adverse events, with inconsistent findings regarding decreased appetite and diarrhea. Other adverse events, including nausea, as well as discontinuation rates, were comparable between the metformin and control groups (**Supplementary Table S1**).

## Discussion

This systematic review, which included four RCTs and 271 participants, is the first to examine the neurocognitive effects of metformin in patients with schizophrenia. The primary findings are as follows: 1) metformin demonstrated significant superiority over controls in improving neurocognitive function as measured by the MCCB, RBANS, and MMSE, but not the BACS; 2) metformin combined with TAU did not significantly improve total psychopathology; and 3) metformin is safe and well-tolerated for enhancing neurocognitive function, though further studies with larger sample sizes are warranted.

In this review, 75% of the included RCTs (20–22) reported that metformin outperformed controls in improving neurocognitive function. However, one study (23) did not observe significant improvements as assessed using the BACS. This inconsistency may stem from the differing assessment tools employed across studies. Evidence suggests that the MCCB has become the gold standard for evaluating neurocognitive impairments in schizophrenia due to its comprehensive scope and strong psychometric properties. Specifically developed for schizophrenia populations, the MCCB has been extensively validated across diverse settings and populations (44). In contrast, the BACS, while more concise, may not fully capture the breadth of neurocognitive deficits as effectively as the MCCB (45). Such inconsistencies have also been reported in previous studies examining neurocognitive function in schizophrenia using different assessment tools. For instance, Hei et al. (46) found that adjunctive sulforaphane significantly improved working memory and verbal learning in schizophrenia, as measured by the MCCB and Hopkins Verbal Learning Test, but showed no significant effects on other measures, including the BACS. Collectively, these findings highlight the importance of employing standardized neurocognitive test batteries, such as MCCB, to ensure consistent and robust assessments. Future research should focus on elucidating the neurocognitive effects of metformin in patients with schizophrenia using standardized tools and well-powered study designs (47).

A recent meta-analysis (6) highlighted that, beyond improving neurocognitive function, metformin also provides benefits for weight management and metabolic syndrome in patients with schizophrenia. Furthermore, several studies have demonstrated metformin's potential to enhance neurocognitive function in other neurological conditions, including Parkinson’s disease (48), Alzheimer’s disease (49), and pediatric brain tumors (50). In the broader context of pharmacological treatments for neurocognitive dysfunction, drugs such as sulforaphane (46), erythropoietin (51), and huperzine A (52) also show promise in improving neurocognitive outcomes. However, no head-to-head studies have compared the neurocognitive effects of metformin with sulforaphane or erythropoietin in adults with schizophrenia. As a result, the relative efficacy of these drugs for neurocognitive enhancement remains unclear.

The mechanisms underlying metformin’s role in improving neurocognitive function in schizophrenia are not yet fully understood. One plausible explanation involves its ability to improve insulin resistance (6, 53, 54), a key pathophysiological factor associated with neurocognitive impairment (55). Insulin resistance has also been linked to weight gain (25), and metformin’s weight-reduction effects may further contribute to neurocognitive improvements. Studies have demonstrated that weight loss is associated with better neurocognitive outcomes, particularly in individuals with obesity or metabolic syndrome (56, 57). Furthermore, metformin’s positive effects on other metabolic indicators, such as lipid levels and glycemic control (25, 58), may also play a role in indirectly enhancing neurocognitive function. Besides its impact on metabolism, metformin decreases inflammation by altering pro-inflammatory cytokines, potentially enhancing neurocognitive function. In an animal study, metformin reduced the levels of pro-inflammatory cytokines like interleukin (IL)-1β, potentially aiding in the enhancement of spatial memory in diabetic animals (59). Furthermore, elevated levels of peripheral cytokines such as IL-1β are found in some patients with schizophrenia and are associated with neurocognitive impairment (60). This multifaceted approach underscores the potential of metformin in the integrated management of neurocognitive impairment in patients with schizophrenia.

Two RCTs reported mixed findings regarding the effects of metformin on decreased appetite and diarrhea compared with controls (20, 23), while both groups exhibited similar rates of discontinuation and other adverse events. Consistent with prior research, daily metformin at recommended dosages (61), is considered notably safe for both short-term and long-term use, with no significant adverse effects reported (6, 20, 24). Gastrointestinal side effects, such as diarrhea and decreased appetite, were the most commonly reported adverse events in this systematic review. These effects, typically observed at the onset of therapy, can be mitigated by lowering the dose, implementing gradual dose titration, or taking the medication with meals (61). Metformin has also demonstrated safety and tolerability in patients with schizophrenia (25), Alzheimer’s disease (49), pediatric brain tumors (50), and bipolar depression (62). However, prolonged use of metformin has been associated with reduced vitamin B12 levels and, in some cases, biochemical B12 deficiency (63, 64). Therefore, it is advisable for patients with schizophrenia receiving metformin to undergo regular monitoring of blood lactate, serum B12, and folate levels (63).

Several limitations of this systematic review should be acknowledged. First, the included RCTs utilized diverse methodologies, including four distinct assessment tools to measure neurocognitive function. This heterogeneity prevented the conduct of a meta-analysis. Importantly, it is different to determine the superiority of metformin over controls in improving a specific neurocognitive function. Second, this review included only four RCTs with a small sample size (n=271, ranging from 30 to 100 participants), necessitating caution in interpreting the findings. Third, the four RCTs focused on the use of metformin in adult patients with schizophrenia, limiting the generalizability of the findings to broader populations. Fourth, this systematic review of metformin for neurocognitive dysfunction in schizophrenia is not registered. Finally, the RCTs included participants with varying conditions, such as schizophrenia combined with glucose metabolism disorder, type 2 diabetes or prediabetes, which may have influenced the outcomes.

## Conclusions

Preliminary evidence suggests that metformin may have beneficial effects on neurocognitive function in schizophrenia. However, to validate these findings, future research should focus on conducting large-scale, double-blind, and high quality RCTs.

## Data Availability

The original contributions presented in the study are included in the article/**Supplementary Material**. Further inquiries can be directed to the corresponding authors.

## Supplementary material

Supplementary Table 1 Metformin for neurocognitive dysfunction in schizophrenia: discontinuation rate and adverse events.
