# Supplementary material for: Metformin for neurocognitive dysfunction in schizophrenia: a systematic review
Source: Front Psychiatry. 2025 Jan 20;15:1540153. doi: 10.3389/fpsyt.2024.1540153 (PMC11788895; doi:10.3389/fpsyt.2024.1540153)
Supplement: Supplementary Table 1 — Metformin for neurocognitive dysfunction in schizophrenia: discontinuation rate and adverse events. [file Table1.docx]

Supplemental Table 1**. Metformin for neurocognitive dysfunction in schizophrenia: discontinuation rate and adverse events.**

| **Study** | **Discontinuation rate (n, %)** | **Metformin group (n, %)** | **Control group (n, %)** | **Findings^a^** |
| --- | --- | --- | --- | --- |
| Agarwal et al., 2021 (Canada) | 8 (26.7) | 7 (33.3) | 1 (11.1) | NS |
| Shao et al., 2023 (China) | 13 (18.1) | 9 (18.8) | 4 (16.7) | NR |
| Wang et al., 2019 (China) | NR | NR | NR | NA |
| Xiong et al., 2021 (China) | NR | NR | NR | NA |
| **Study** | **Adverse effects** | **Metformin group (n, %)** | **Control group (n, %)** | **Findings^a^** |
| Agarwal et al., 2021 (Canada) | Abdominal bloating | 3 (14.3) | 0 (0) | NR |
|  | Abdominal pain | 2 (9.5) | 1 (11.1) | NS |
|  | Acid reflux | 2 (9.5) | 0 (0) | NR |
|  | Constipation | 4 (19.0) | 4 (44.4) | NS |
|  | Death | 1 (4.8) | 0 (0) | NR |
|  | Decreased appetite | 2 (9.5) | 2 (22.2) | NS |
|  | Depression | 0 (0) | 1 (11.1) | NR |
|  | Diarrhea | 8 (38.1) | 7 (77.8) | P < 0.05 |
|  | Difficulty concentrating | 0 (0) | 1 (11.1) | NR |
|  | Dizziness | 3 (14.3) | 3 (33.3) | NS |
|  | Dysgeusia | 2 (9.5) | 0 (0) | NR |
|  | Fatigue | 1 (4.8) | 2 (22.2) | NS |
|  | Functional dyspepsia | 1 (4.8) | 2 (22.2) | NS |
|  | Headache | 1 (4.8) | 1 (11.1) | NS |
|  | Increase in psychosis | 1 (4.8) | 0 (0) | NR |
|  | Increased appetite | 1 (4.8) | 1 (11.1) | NS |
|  | Influenza-like illness | 1 (4.8) | 1 (11.1) | NS |
|  | Irritated/bad mood | 1 (4.8) | 0 (0) | NR |
|  | Light headedness | 1 (4.8) | 1 (11.1) | NS |
|  | Muscle spasm | 2 (9.5) | 0 (0) | NR |
|  | Myalgia (muscle pain) | 1 (4.8) | 0 (0) | NR |
|  | Myasthenia (muscle weakness) | 0 (0) | 1 (11.1) | NR |
|  | Nausea | 9 (42.9) | 5 (55.6) | NS |
|  | Palpitations | 0 (0) | 1 (11.1) | NR |
|  | Paraesthesias | 2 (9.5) | 0 (0) | NR |
|  | Pregnancy | 1 (4.8) | 0 (0) | NR |
|  | Pruritus | 1 (4.8) | 0 (0) | NR |
|  | Pulmonary embolism | 1 (4.8) | 0 (0) | NR |
|  | Rash | 0 (0) | 1 (11.1) | NR |
|  | Vomiting | 3 (14.3) | 2 (22.2) | NS |
|  | Xerostomia | 2 (9.5) | 0 (0) | NR |
| Shao et al., 2023 (China) | Abnormal hemogram | 2 (4.4) | 0 (0) | NS |
|  | Abnormal liver function | 2 (4.4) | 3 (12.5) | NS |
|  | Decreased appetite | 15 (33.3) | 0 (0) | P < 0.05 |
|  | Diarrhea | 1 (2.2) | 0 (0) | NS |
|  | Nausea and vomit | 2 (4.4) | 0 (0) | NS |
|  | Somnolence | 3 (6.7) | 2 (8.3) | NS |
|  | Tachycardia | 3 (6.7) | 1 (4.2) | NS |
| Wang et al., 2019 (China) | NR | NR | NR | NA |
| Xiong et al., 2021 (China) | NR | NR | NR | NA |
| **^a^** The differences between metformin groups and control groups at the treatment endpoints.  Abbreviations: NA=not applicable; NR=not reported; NS=not significant. | | | | |
